# Modification of Epigenetic Patterns in Low Birth Weight Children: Importance of Hypomethylation of the *ACE* Gene Promoter

**DOI:** 10.1371/journal.pone.0106138

**Published:** 2014-08-29

**Authors:** Marina Rangel, Jéssica Cassilla dos Santos, Paula Helena Lima Ortiz, Mario Hirata, Miriam Galvonas Jasiulionis, Ronaldo C. Araujo, Daniela Filippini Ierardi, Maria do Carmo Franco

**Affiliations:** 1 Nephrology Division, School of Medicine, Federal University of São Paulo, São Paulo, Brazil; 2 Molecular Biology Laboratory. Dante Pazzanese Institute of Cardiology, São Paulo, Brazil; 3 School of Pharmaceutical Sciences, University of São Paulo, São Paulo, Brazil; 4 Pharmacology Department, Federal University of São Paulo, São Paulo, Brazil; 5 Biophysics Department, Federal University of São Paulo, São Paulo, Brazil; 6 Ben May Department for Cancer Research, University of Chicago, Chicago, Illinois, United States of America; University of Southampton, United Kingdom

## Abstract

There is a growing body of evidence that epigenetic alterations are involved in the pathological mechanisms of many chronic disorders linked to fetal programming. Angiotensin-converting enzyme (ACE) appears as one candidate gene that brings new insights into the epigenetic control and later development of diseases. In this view, we have postulated that epigenetic modifications in the *ACE* gene might show different interactions between birth weight (BW), blood pressure levels, plasma ACE activity and ACE I/D polymorphism. To explore this hypothesis, we performed a cross-sectional study to evaluate the DNA methylation of 3 CpG sites using pyrosequencing within the *ACE* gene promoter of peripheral blood leukocytes from 45 LBW children compared with 70 NBW children. Our results have revealed that LBW children have lower methylation levels (*P<*0.001) in parallel with a higher ACE activity (*P = *0.001). Adjusting for prematurity, gender, age, body mass index, and family history of cardiovascular disease did not alter these findings. We have also performed analyses of individual CpG sites. The frequency of DNA methylation was significantly different at two CpG sites (site 1: nucleotide position +555; and site 3: nucleotide position +563). In addition, we have found a significant inverse correlation between degree of DNA methylation and both ACE activity (*P*<0.001) and systolic blood pressure levels (*P*<0.001). We also observed that the methylation level was significantly lower in LBW children who are carriers of the DD genotype compared to NBW children with DD genotype (*P*<0.024). In conclusion, we are able to demonstrate that the hypomethylation in the 3 CpG sites of *ACE* gene promoter is associated with LBW in 6 to 12 year-old children. The magnitude of these epigenetic changes appears to be clinically important, which is supported by the observation that discrete changes in DNA methylation can affect systolic blood pressure and ACE protein activity levels.

## Introduction

Epigenetic programming refers to alterations in gene expression which are not explained by changes in the DNA sequence itself. The mechanisms involved in these modifications comprise several levels of gene regulation, which include DNA methylation, histone modifications and RNA-based mechanisms [1], [3]. It has been suggested that epigenetic programming is susceptible to a number of insults that occur *in utero*, leading to long-term changes in phenotype [4]–[6].

The role of DNA methylation has been studied recently in experimental models of fetal programming, suggesting that epigenetic aspects are an important link between the intrauterine environment and the risk for developing chronic diseases in later life [7]–[12]. It is known that DNA methylation is largely established during fetal or early postnatal life. DNA methylation is the most stable epigenetic mark that influences the maintenance of genome integrity, silencing of imprinted genes, X chromosome inactivation and gene expression [1]–[6]. DNA methylation occurs primarily at cytosines in cytosine-guanine dinucleotides (CpG) [13], [14]. About 80% of CpG dinucleotides in mammalian genomes are methylated. On the other hand, there are regions of DNA sequence that are very rich in CpG dinucleotides, termed CpG islands which primarily occur in gene promoter regions. It is known that during active gene transcription the CpG islands are unmethylated. An interesting characteristic of many genes (∼60%) is that they have a CpG island at the 5′ end of the promoter region which is important for transcriptional regulation [13], [14].

The angiotensin-converting enzyme (ACE), a member of the renin-angiotensin system (RAS) has been investigated extensively as a key gene for cardiovascular disease (CVD) [15]–[18]. The *ACE* gene has been cloned and found to be characterized in humans by a major polymorphism consisting of the insertion (I) or deletion (D) of a 287-bp sequence within intron 16. It has been demonstrated that the D allele and the DD genotype are associated with elevated levels of ACE and a higher risk of adverse cardiovascular events and end-organ damage [15], [16], [18]. A recent study from our group proposed an involvement of ACE insertion/deletion (I/D) polymorphisms in the mechanisms of fetal programming of hypertension [19]. We have shown that low birth weight (LBW) children had a higher D allele frequency than normal birth weight (NBW) children. When analyzed by quartiles of SBP or ACE activity, we found a greater frequency of LBW children who were carrying the DD genotype in the highest quartiles of these parameters, whereas the NBW children tended to be in the lowest quartile. It is intriguing that the ACE I/D polymorphism is responsible for 40%–50% of the ACE serum concentration variation, the remaining half could be determined by epigenetic patterns [20]. It is acknowledged that the promoter region of the *ACE* gene is characterized by a large number of CpG sites [21]. An alternative approach to understanding these ACE-related alterations could be to assess the methylation status in the gene promoter region.

In this context, fetal programming appears to involve epigenetic modifications in response to alterations of the intrauterine environment. These modifications are laid down largely during early development and remain relatively fixed over a lifetime [4], [6], [7]. Thus, it is conceivable that the epigenetic modification of the ACE gene might show different interactions between birth weight, blood pressure levels, plasma ACE activity and ACE I/D polymorphism.

Although it is known that ACE may be detected in plasma, the major part of ACE exists on the vascular endothelium, in non-endothelial parenchymal cells, and on circulating leukocytes [22]. It has been reported that ACE expression on peripheral blood mononuclear cells may contribute to local RAS activation and to subsequent progression of the CVD [22]. Given this, we investigated DNA methylation in the regulatory region of the ACE gene in peripheral blood leukocyte of 115 children with both normal birth weight and low birth weight.

## Methods

The children we studied are a subsample of one cohort from a recently published cross-sectional study by Ajala et al. [19]. Briefly, a total of 167 children were selected (83 girls and 84 boys). Among these children, 60 (31 girls and 29 boys) were identified as born with a low birth weight (LBW children: BW≤2.5 Kg), and 107 (52 girls and 55 boys) with a normal birth weight (NBW children: BW≥3.0 Kg) [17]. To carry out this study, we excluded 52 children (LBW: 15 and NBW: 37) because of inadequate amounts of blood samples or DNA for evaluation of the *ACE* gene promoter methylation. One hundred and fifteen children, aged from 6 to 12, remained eligible for this study. Medical history was taken and physical examination was performed in order to exclude severe medical disorders. No child had any clinical signs of hypertension, endocrinopathy, renal disease or chronic illness. After the children were fasted overnight, blood samples were collected by venipuncture of a forearm vein under standardized conditions. The birth weight (BW) or gestational age data were derived from maternal recall and later were cross-checked with records obtained from the hospitals where the children were born. This study was approved by the Research Ethics Committee of the Federal University of São Paulo (UNIFESP), and written informed consent was obtained from the parents of the children enrolled in the study. The procedures followed were in accordance with our institutional guidelines.

### Clinical Variables

Height was measured to the nearest 0.1 cm with a stadiometer and weight to the nearest 0.1 kg with a mechanical medical scale. Abdominal circumference was measured using a flexible tape to the nearest 0.5 cm midway between the lower rib and the top of the iliac crest. Systolic (SBP) and diastolic (DBP) blood pressures were measured by auscultation after the child had remained seated for 10 min. Three measurements were made at 2-min intervals, and the mean value was used in the analysis. The same nurse, blinded to the clinical data, examined and evaluated all 115 children.

### Plasma ACE Activity

For plasma ACE activity, heparin tubes were centrifuged and the plasma samples were stored at −80°C until analysis. The plasma ACE activity was measured using Hippuryl-histidine-leucine (HHL) as a substrate by a fluorometric method [23]. Briefly, 10 µl plasma samples were incubated with 490 µl of assay buffer containing 5 mM Hip-His-Leu in 0.4 M sodium borate buffer and 0.9 M NaCl for 15 min at 37°C. The reaction was stopped by the addition of 1.2 ml of 0.34 N NaOH. The product, His-Leu, was measured fluorimetrically (365-nm excitation and 495-nm emission) after the addition of 100 µl of *o*-phthaldialdehyde (20 mg/ml) in methanol, which was followed 10 minutes later by the addition of 200 µl 3 N HCl and centrifuged (800 *g* for 5 minutes) at room temperature. A standard curve of 0.5 to 20 nmol His-Leu/tube was prepared for each assay. Enzyme activity is reported as nmol/mL/min. Assays were carried out under conditions that provided constant velocity and constant enzyme-specific activity. To correct for the intrinsic fluorescence of the plasma, time zero blanks (T_0_) were prepared by reversing the order of the addition of enzyme and NaOH. The T_0_ blanks could be used because the relative fluorescence of plasma (without substrate) or substrate (without plasma) was constant under the conditions of the assay [23]. All assays were performed in triplicate by the same researcher, who used a blind analysis technique.

### DNA Isolation and Bisulfite Treatment

For DNA isolation, EDTA tubes were centrifuged and the peripheral blood leukocyte (buffy coat) were extracted and stored at −80°C until analysis. Isolation of genomic DNA was performed using the QIAmp DNA Mini kits (Qiagen, Hilden, Germany). The integrity of the isolated DNA was checked on a 1% agarose gel. Bisulfite treatment of up to 1 µg of DNA was performed using an EZ DNA Gold Methylation kit (Zymo Research, U.S.A.) according to the manufacturer’s instructions.

### Methylation Analysis Using Pyrosequencing

Pyrosequencing analysis was carried out for 3 CpG sites in intron 1 of the ACE promoter using a PyroMark Q24 System (Qiagen, Hilden, Germany). We used the predesigned methylation assay from Qiagen (Hilden, Germany). The 3 CpG sites chosen are part of the CpG island that includes: the promoter region, the 5′UTR, the first exon and the first intron defined in the UCSC genome browser (Figure S1). Details of the predesigned methylation assay used, including primer sequences, are provided in Table S1. After bisulfite conversion, genomic DNA (20 ng) was amplified by real time-PCR (Rotor Gene Q, Qiagen, Hilden, Germany). The PCR reactions were conducted using the PyroMark PCR kit (Qiagen, Hilden, Germany) according to the manufacturer’s protocol. PCR conditions were: 95°C for 15 min, followed by 45 cycles of 94°C for 30 s, 56°C for 30 s, 72°C for 30 s, and, finally, 72°C for 10 min. Pyrosequencing methylation analyses were conducted using the PyroMark Q24 (Qiagen, Hilden, Germany) according to the manufacturer’s protocol. The level of methylation was analyzed using PyroMark Q24 2.0.6 Software (Qiagen, Hilden, Germany), which calculates the methylation percentage (mC/(mC+C)) for each CpG site, allowing quantitative comparisons (mC is methylated cytosine, C is unmethylated cytosine). The results were expressed by the sum of the methylation percentage for each CpG site (nucleotide positions +555, +561, +563) of each child. To compare the methylation levels (%) among BW groups, we used the average of the three CpG sites. To verify the accuracy of the analysis, each run included a negative control without template and a control DNA from the EpiTect PCR Control DNA Set (Qiagen, Hilden, Germany) that had contained both bisulfite converted 100% methylated as well as unmethylated DNA as positive control and unconverted unmethylated DNA as a negative control.

### Statistical Analysis

All continuous variables were examined for normality with the Kolmogorov-Smirnov test. Categorical variables were compared using the χ^2^ test. Student’s *t* test was used to compare the mean values of continuous variables between BW groups. Analysis of covariance (ANCOVA) was used to compare the mean values of ACE protein activity, blood pressure levels and DNA methylation between BW groups, adjusting for prematurity, gender distribution, age, and family history of CVD. Mann–Whitney *U* test was further calculated to analyze the differences in DNA methylation between BW groups at a particular I/D genotype, while the relationship between DNA methylation between I/D genotype was assessed using Kruskal-Wallis test. Haploview software was used to analyze the ACE I/D alleles and to determine their Hardy-Weinberg (HW) equilibrium status. The values of continuous variables are expressed as the mean ± standard error of the mean (SEM) or median value and the interquartile range (IQR). Pearson’s correlation coefficients were used to investigate associations between variables, before and after adjusting for confounding factors. Statistical tests were two-tailed, and the significance level was set at *P*<0.05. Statistical analyses were conducted using SPSS version 13.0.

## Results

We investigated the DNA methylation of 3 CpG sites in the gene promoter of the *ACE* in peripheral blood leukocyte from 70 NBW children (41 boys and 29 girls) and 45 LBW children (20 boys and 25 girls). There were no significant differences between the birth weight groups regarding gender, age, or family history of CVD (Table 1). No difference in weight, height or body mass index (BMI) was found between the two groups of children. In contrast to NBW children, abdominal circumference was greater in LBW children (Table 1). Children with a history of LBW showed higher blood pressure levels when compared with those born with a normal weight; also, ACE protein activity levels were significantly elevated in these children (Table 1).

**Table 1:** Main Characteristics of the Study Population.

		NBW Children(n = 70)	LBW Children(n = 45)	*P* Values
Birth weight (g)		3392.9±27.2	2292.3±23.9	<0.001
Prematurity				
	Yes (%)	(0)	(16)	<0.001
	No (%)	(100)	(84)	
Gender				
	Girls (%)	(41)	(56)	0.138
	Boys (%)	(59)	(44)	
Age (years)		8.8±0.19	9.3±0.26	0.193
Weight (kg)		31.2±0.79	33.8±1.19	0.077
Height (cm)		134.8±1.23	138.7±1.58	0.055
Abdominal circumference (cm)		58.4±0.68	61.7±1.15	0.016
BMI (kg/m^2^)		16.9±0.21	17.4±0.35	0.217
SBP (mmHg)		94±0.85	101±1.04	<0.001
DBP (mmHg)		66±0.75	69±0.86	0.035
Family history of CVD				
	Yes (%)	(12)	(16)	0.683
	No (%)	(88)	(84)	
ACE activity (nmol/mL/min)		75.6±2.44	90.9±3.22	<0.001
DNA methylation in *ACE* gene (%)		7.0±0.19	5.3±0.23	<0.001

Values are expressed as mean ± SEM. Data was analyzed using two-tailed Student’s t test. BMI, body mass index; CVD, Cardiovascular disease; SBP, systolic blood pressure; DBP, diastolic blood pressure.

We assessed the DNA methylation of 3 CpG sites (nucleotide positions +555, +561, +563) using pyrosequencing within the *ACE* gene promoter. In the LBW children, there was a decreased in methylation levels (mean: 5.4±0.28% in average over all 3 CpG sites) when compared to NBW children (mean: 6.8±0.19%). Among the LBW children, 58% were below the 50th percentile for DNA methylation in *ACE* gene promoter and 18% remained below the 10th percentile. In contrast, among NBW children, 30% were below the 50th percentile, and no child was below the 10th percentile (*P* = 0.025).

**Figure 1:**
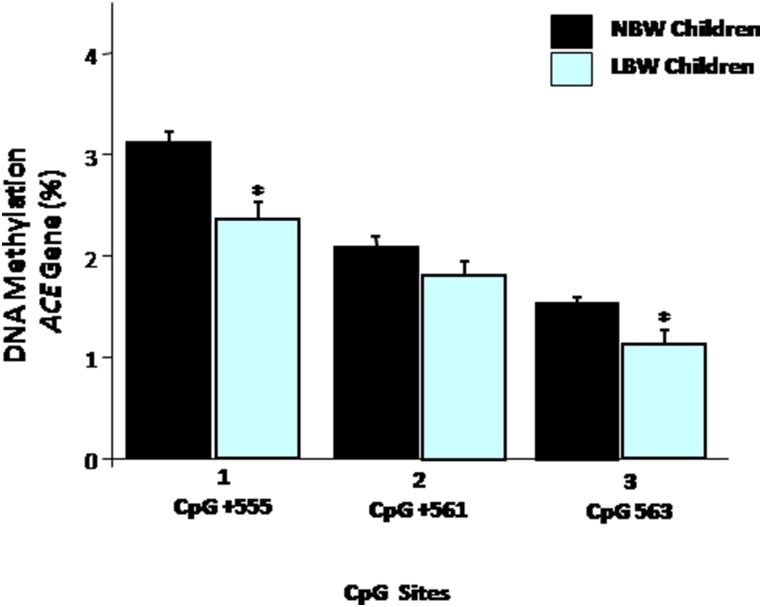
Bar graphs represents mean DNA methylation at individual CpG sites **within the** ***ACE*** gene promoter in NBW and LBW children. The error bars indicate the standard error of mean and statistical significance was calculated using *two-tailed Student’s t Test*. *P<0.05 NBW children vs. LBW children.

To extend our data, we performed analyses of individual CpG sites. The frequency of DNA methylation was significantly different at two CpG sites (site 1: nucleotide position +555; and site 3: nucleotide position +563) (Figure 1). These results have also revealed that LBW children have lower levels of DNA methylation at CpG site 1 (*P* = 0.001) and 3 (*P* = 0.009) when they are compared with NBW children. No significant difference at CpG site 2 (nucleotide position +561) was observed (*P* = 0.136).

It is known that potential confounding variables can contribute to these alterations on blood pressure levels, ACE protein activity levels and DNA methylation. For these reasons, we adjusted our data by prematurity, gender, age, BMI, and family history of CVD. In the ANCOVA model, the adjustment for these confounding factors did not alter the results (Table 2). We carried out correlation analyses to clarify the relationship between DNA methylation in the *ACE* gene with blood pressure levels and ACE protein activity. In the first model, we found that SBP and ACE protein activity levels were inversely related to the percentage of DNA methylation (Table 3). Model 2 indicated that this negative relationship persists in the presence of some important confounding factors–prematurity, gender, age and family history of CVD–whereas the correlation coefficient between ACE protein activity levels and DNA methylation improved (Table 3). In contrast, the coefficient for SBP and ACE protein activity worsened their significance in Model 3, when birth weight data were included in the final model (Table 3).

**Table 2:** Blood Pressure Levels, ACE Activity and DNA Methylation in *ACE* Gene in Adjusted Covariance Model for Prematurity, gender, age, BMI and Family History of Cardiovascular Disease.

	NBW Children(n = 70)	LBW Children (n = 45)	*P* Values
SBP (mmHg)	94 (91.6 to 95.4)	101 (99.2 to 103.5)	<0.001
DBP (mmHg)	66 (64.2 to 66.9)	69 (67.5 to 71.0)	0.002
ACE activity (nmol/mL/min)	76.1 (71.0 to 81.2)	90.2 (83.7 to 96.6)	0.001
DNA methylation in *ACE* gene (%)	6.8 (6.4 to 7.2)	5.1 (4.7 to 5.8)	<0.001

Values are adjusted for prematurity, gender, age, BMI, and family history of CVD. All values are expressed as means (95% confidence interval). Data were analyzed using ANCOVA. SBP, systolic blood pressure; DBP, diastolic blood pressure.

**Table 3:** Linear Correlation between of DNA Methylation in *ACE* Gene with ACE Activity and Blood Pressure Levels.

	DNA Methylation in ACE Gene					
	Model 1		Model 2		Model 3	
	*r*	*P Value*	*r*	*P Value*	*r*	*P Value*
ACE activity (nmol/mL/min)	–0.430	<0.001	–0.442	<0.001	–0.362	<0.001
SBP (mmHg)	–0.354	<0.001	–0.345	<0.001	–0.206	0.031
DBP (mmHg)	–0.129	0.168	–0.149	<0.109	–0.071	0.462

Data were analyzed using Pearson’s correlation coefficients. SBP, systolic blood pressure; DBP, diastolic blood pressure. Model 1: no adjustment (crude model); Model 2: adjustment by prematurity, gender, age and family history of CVD and; Model 3: adjustment by birth weight, prematurity, gender, age and family history of CVD.

**Figure 2:**
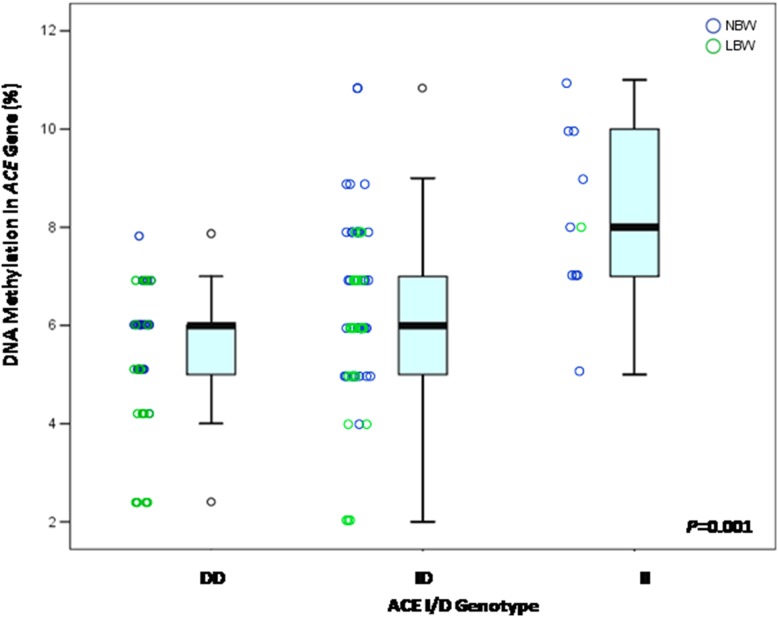
Box plot of median DNA methylation of 3 CpG sites within the *ACE* gene promoter among ACE I/D genotypes, displayed with the scatter plot of raw data. The inside each box represents the median, and the lower and upper edges of the boxes represents the 25^th^ and 75^th^ percentiles, respectively, and upper and lower lines outside the boxes represent minimum and maximum values (error bars). The differences of DNA methylation among ACE I/D genotypes is statistically significant (*P* = 0.001; *Kruskal-Wallis Test*).

**Figure 3:**
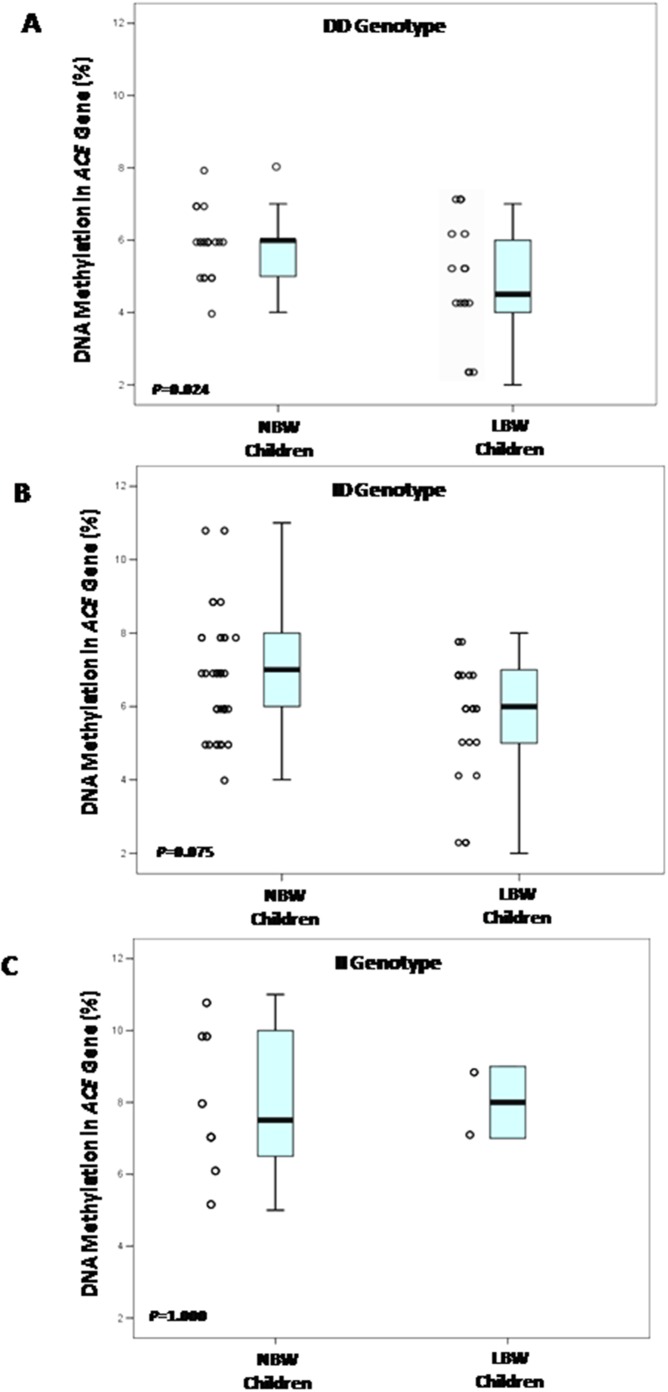
Box plot of median DNA methylation of 3 CpG sites within the *ACE* gene promoter among BW groups stratified by ACE I/D genotypes, displayed with the scatter plot of raw data. (A) Box plot demonstrates median DNA methylation in 42 children stratified by DD genotype (P = 0.024; *Mann–Whitney Test*); (B) Box plot demonstrates median DNA methylation in 63 children stratified by ID genotype (P = 0.075; *Mann–Whitney Test*); (C) Box plot demonstrates median DNA methylation in 10 children stratified by II genotype (P = 1.000; *Mann–Whitney Test*). The inside each box represents the median, and the lower and upper edges of the boxes represents the 25^th^ and 75^th^ percentiles, respectively, and upper and lower lines outside the boxes represent minimum and maximum values (error bars).

We investigated the interaction of ACE I/D polymorphism on DNA methylation levels. Among the children who were evaluated, 10 (0.09) were homozygous for the I allele (II) (three LBW children and seven NBW children), 63 (0.54) were heterozygous (ID) (22 LBW children and 41 NBW children), and 42 (0.37) were homozygous for the D allele (DD) (20 LBW children and 22 NBW children). There were significant differences in the DNA methylation among the DD, ID and II genotypes (*P = *0.001; *Kruskal-Wallis* test) (Figure 2). As shown in Figure 3, the methylation level of the 3 CpGs sites in the ACE gene promoter was significantly lower in LBW children who are carriers DD genotype, with a median value of 4.5% (IQR: 2%–7%) compared to 6% (IQR: 4%–8%, *P * =  0.024) in NBW children with DD genotype. On the other hand, there were no significant differences in the DNA methylation levels among BW groups in the ID (NBW - median value 7% and IQR: 4%–11%; LBW - median value 6% and IQR: 2%–8%, *P* = 0.075) or II genotype (NBW-median value 8% and IQR: 5%–11%; LBW-median value 8% and IQR: 7%–9%, *P* = 1.000) (Figure 3A, B and C).

## Discussion

This study has demonstrated that the DNA methylation status in 3 CpG sites from the *ACE* gene promoter was decreased in LBW children. Moreover, this hypomethylation occurs simultaneously with an increase in the ACE protein activity and high blood pressure levels in these children. Our data further demonstrate that SBP levels and ACE protein activity were inversely correlated with the degree of DNA methylation. Therefore, it seems probable that the epigenetic regulation of *ACE* gene promoter by hypomethylation could be a mechanism whereby this enzyme might contribute to the development of chronic diseases related to fetal programming.

An extensive body of studies has shown that LBW is related to a greater risk of cardiometabolic comorbities, such as hypertension, insulin resistance, dyslipidemia and obesity [24]–[27]. The underlying mechanisms are still unclear, but genetic and epigenetic factors associated with both fetal growth and increased risk of cardiometabolic disease may be involved. It is known that RAS is more activated in the neonatal period and has an important role in fetal development and growth [28]. In addition, the hyperactivity of this system has been implicated in the development of hypertension and other cardiometabolic disorders [29]–[31]. Previous studies have revealed that LBW is associated with the modification of some elements of the RAS. Miyawaki et al. [32] showed that plasma angiotensin II (AII) levels in early postnatal life were higher in LBW than in NBW infants. Another study has shown that ACE protein activity was inversely correlated with BW in 3 month of age children [33]. We have previously reported that AII concentration and ACE protein activity were increased in LBW children [19], [34]. Moreover, we found that LBW children have higher frequency of the ACE D allele. Our present findings are not only consistent with these previous data but also extended them. Here we are able to demonstrate that hypomethylation in 3 CpG sites of the *ACE* gene promoter is associated with LBW in 6 to 12 year-old children, even after adjustment for potential confounders. In accordance with these findings, 58% of the LBW children were below the 50th percentile for DNA methylation in the *ACE* gene promoter and 18% remained below the 10th percentile. The magnitude of this epigenetic modification appears to be clinically important, which is supported by the observation that a discrete change in DNA methylation correlates with both SBP and ACE protein activity levels.

Epigenetic marks are an important mechanism by which heritable changes in gene activity and expression occur without alteration in the DNA sequence [2]. It is known that epigenetic modifications, such as DNA methylation, can be determined during fetal development and remain relatively fixed over a lifetime and through generations [4], [6]. In this context, the Hunger Winter Families Study demonstrated that individuals whose parents had been exposed to famine during the preconception period had a reduction in the DNA methylation of *insulin-like growth factor* -*2 (IGF-2)* gene in old age [35], [36].

In human, the existence of CpG sites in the *ACE* gene underscores the high susceptibility of this gene to methylation and consecutive modulation of the ACE expression. DNA methylation and histone acetylation have been shown to influence the regulation of somatic ACE [21]. Rivière et al. [21] found that the methylation status of two CpG sites (−562/−244 and −202/−216), along with the acetylation status of histones, strongly influences the activity of the *ACE* gene promoter in driving transcription in a cell type-specific manner [21]. In this respect, we evaluated 3 CpG sites within a CpG island which includes the promoter region, the 3′UTR, the first exon and the first intron. Although the three selected CpG sites are not located within the two CpG sites regions (−562/−244 and −202/−216) previously defined as key promoter regions, they are located at the first intron that is still a region defined as an expression regulation controlling region. Moreover, these three CpG sites are not within the ALU element region subjected to the I/D mutation. In the present study, the analysis of the three CpG sites together indicated that the average DNA methylation of the *ACE* gene promoter was lower in LBW children when compared to NBW subjects. Additionally, when analyzing separately, we found difference at two CpG sites (site 1: nucleotide position +555; and site 3: nucleotide position +563). These results also revealed that LBW children had lower levels of DNA methylation at CpG sites 1 and 3, when they were compared to NBW children. It is noteworthy that the mean methylation levels of three CpGs in the *ACE* gene are inverse correlated with ACE protein activity and SBP levels. Similar results have been found after adjustment for prematurity, gender, age and family history of CVD. When birth weight was included in the final model, the magnitude of these correlations decreased, suggesting that the strength of the associations occurred due to birth weight differences among children. It may be possible that the hypomethylation observed in these two specific CpG sites, within the *ACE* gene promoter, directly contributes to a higher ACE protein activity and could predispose LBW children to undesirable outcomes later in life, such as elevated SBP levels observed in these children.

Additional molecular mechanisms related to the *ACE* gene should also be considered. If changes in the DNA methylation levels of three CpGs in the *ACE* gene can promote higher ACE protein activity, interactions with functional ACE I/D polymorphism can also have a part to play. A recent study from our group investigated possible interactions between the ACE I/D polymorphism and birth weight as well as their effects on RAS components and blood pressure levels in healthy children. We concluded that there was a positive trend connecting the DD genotype with both high ACE protein activity and SBP levels in LBW children [19]. In the present study, the presence of homozygous DD genotype resulted in a decrease of DNA methylation in LBW children. Under this context, there is a possible interaction between ACE I/D polymorphism and DNA methylation of three CpGs sites in the *ACE* gene promoter in LBW children. We have to consider that without prior knowledge of the potential role of this interaction, it is extremely difficult to prove that it is present for a given LBW phenotype. The demonstration of synergistic or interactive effects from the epigenetic regulation of the *ACE* gene and I/D polymorphism, would not only strongly reinforce the likelihood of their pathophysiological involvement, but also help us to understand the complexity of the genetic architecture.

We are aware that our conclusions are limited. The cross-sectional nature of the study limits our possibilities of empirically examining the extent and direction of causality between birth weight, DNA methylation of the *ACE* gene promoter and blood pressure levels. Another methodological consideration was the use of the customized assay which evaluated only 3 CpG sites, which does not include the CpG regions described by Rivière et al. [21]. Finally, our data are representative of a small population (e.g. LBW children versus NBW children) and, thus, cannot be generalized to different populations and contexts. Indeed, we should keep in mind that epigenetic patterns will not only be population-specific, but also cell-, tissue-, and time-specific.

In conclusion, we were able to demonstrate hypomethylation in the 3 CpG sites of *ACE* gene promoter in 6 to 12 year-old LBW children. The magnitude of these epigenetic changes appears to be clinically important, which is supported by the observation that discrete changes in DNA methylation can correlate with systolic blood pressure and ACE protein activity levels. Moreover, the presence of D alleles resulted in a decrease of DNA methylation, suggesting an interaction between epigenetic regulation of the *ACE* gene and I/D polymorphism in our cohort. Large prospective studies are needed to definitively determine the relevance of this epigenetic mechanism on the control of ACE protein activity and blood pressure levels in LBW children.

## Supporting Information

Figure S1 **Schematic representation shows the analyzed CpG sites relative to the ACE promoter region.** Genomic organization of the promoter region consisting of 6 exons (blue boxes), introns (black lines) and the locations of amplicon (red box) evaluated in this study by pyrosequencing. The 3 CpG sites chosen are part of the CpG island that includes: the promoter region, the 5′UTR, the first exon and the first intron defined in the UCSC genome browser. (http://genome.ucsc.edu/cgi-bin/hgTracks?db=hg19&position=chr17%3A61554170-61555789&hgsid=374432715_qhcffVWK3dBfjwbK3jeR19g1yhCD).
(TIF)

Table S1 **Assay Details for Pyrosequencing Analysis and Primer Sequence.**
(DOC)
