# Supplementary figures and images for: Modification of Epigenetic Patterns in Low Birth Weight Children: Importance of Hypomethylation of the ACE Gene Promoter
Source: PLoS One. 2014 Aug 29;9(8):e106138. doi: 10.1371/journal.pone.0106138 (PMC4149513; doi:10.1371/journal.pone.0106138)

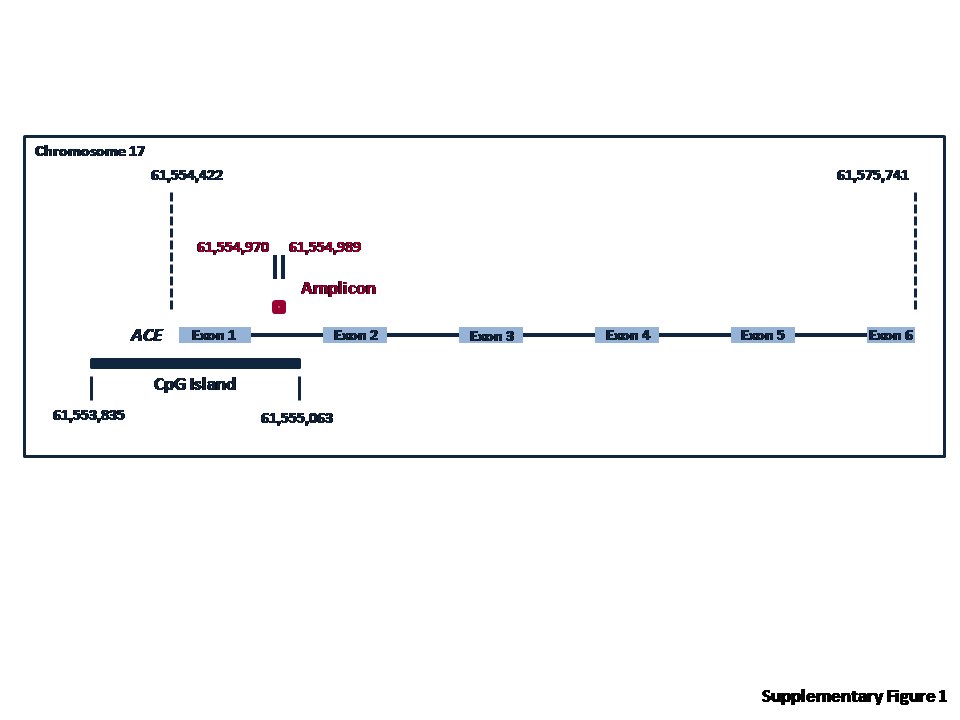

Supplement: Figure S1 — Schematic representation shows the analyzed CpG sites relative to the ACE promoter region. Genomic organization of the promoter region consisting of 6 exons (blue boxes), introns (black lines) and the locations of amplicon (red box) evaluated in this study by pyrosequencing. The 3 CpG sites chosen are part of the CpG island that includes: the promoter region, the 5′UTR, the first exon and the first intron defined in the UCSC genome browser. (http://genome.ucsc.edu/cgi-bin/hgTracks?db=hg19&position=chr17%3A61554170-61555789&hgsid=374432715_qhcffVWK3dBfjwbK3jeR19g1yhCD). (TIF) [file pone.0106138.s001.tif]
