# Supplementary material for: Modification of Epigenetic Patterns in Low Birth Weight Children: Importance of Hypomethylation of the ACE Gene Promoter
Source: PLoS One. 2014 Aug 29;9(8):e106138. doi: 10.1371/journal.pone.0106138 (PMC4149513; doi:10.1371/journal.pone.0106138)
Supplement: Table S1 — Assay Details for Pyrosequencing Analysis and Primer Sequence. (DOC) [file pone.0106138.s002.doc]

**Table S1:** Assay Details for Pyrosequencing Analysis and Primer Sequence.

| Assay Name | Hs_ACE_01_PM (Qiagen) |
| --- | --- |
| Sequence to Analyze | GCTGCC**CGAGAGCGCG**GGGG |
| Number of CpG | 03 |
| | Primer 5’-3’ | | --- | | Included in the Assay |
| Nucleotide dispensation order | TGTCTGTCGTATGAGTCGTCG |
| Amplicon length | 115 bp |
| Amplicon Localization | +548 to +567 |
| Chromosomal Location | Chromosome 17, BP 61554975-61554983 |
| Entrez Gene ID | 1636 |
| Transcripts for this gene | NM_000789, NM_001178057, NM_152830,NM_152831 |
